# Premixed Lidocaine With Fospropofol Disodium for Safety and Clinical Evaluation Regarding Paresthesia Upon Fospropofol Disodium Injection: A Preclinical Experimental Study and a Randomized Controlled Trial

**DOI:** 10.1002/mco2.70670

**Published:** 2026-03-18

**Authors:** Bo Jiao, Xiaolin Xu, Ying Cui, Caiyi Yan, Liyun Deng, Dequan Zhong, Qing Yang, Jiqian Xu, Yi Liu, Xiaohui Sun, Mengqian Xu, Tao Liu, Hui Xu, Xuejiao Tang, Xiaoqin Luo, Peng Liang, Jin Liu, Chan Chen

**Affiliations:** ^1^ Department of Anesthesiology West China Hospital Sichuan University Chengdu Sichuan China; ^2^ Laboratory of Anesthesia and Critical Care Medicine National‐Local Joint Engineering Research Centre of Translational Medicine of Anesthesiology West China Hospital Sichuan University Chengdu Sichuan China; ^3^ Department of Anesthesiology Huaihe Hospital of Henan University Kaifeng China; ^4^ Department of Anesthesiology Ganzi Tibetan Autonomous Prefecture People's Hospital Kangding Sichuan Province China

**Keywords:** clinical trial, experimental study, fospropofol, lidocaine, paresthesia

## Abstract

Fospropofol disodium (fospropofol), a water‐soluble prodrug of propofol, reduces injection pain and anesthetic requirements but frequently causes paresthesia. Intravenous lidocaine has been shown to alleviate dexamethasone‐induced paresthesia, yet its effect on fospropofol‐related symptoms remains uncertain. We combined preclinical and clinical studies, first evaluating the safety and pharmacological changes of fospropofol premixed with lidocaine through in vitro and in vivo experiments and then conducting a randomized controlled trial in adult surgical patients to evaluate whether the lidocaine premixing strategy affects the occurrence of fospropofol‐induced paresthesia. In the preclinical study, the findings indicated that mixture of fospropofol and lidocaine remained physicochemically stable, with faster onset and longer sedation duration compared with fospropofol alone, without additional adverse effects. In the clinical trial, 74 patients received fospropofol dissolved in either 20 mL of normal saline or 0.75% lidocaine and 72 were included in the primary outcome analysis of paresthesia. This adverse reaction occurred in 83.3% of patients in both groups, mainly within 40–60 s after administration. No group differences were observed in plasma inflammatory markers and phosphate; however, phosphate levels increased postadministration in both groups. This study provides important guidance for clinical practice, showing that premixing lidocaine does not effectively alleviate paresthesia induced by fospropofol.

## Graphical abstract

This study explored the effect of lidocaine premixing on fospropofol‐induced paresthesia. While the mixture of fospropofol (FP) and lidocaine (LD) proved chemically stable and safe in preclinical tests, clinical results from randomized controlled trial showed no reduction in paresthesia in FP+LD group compared with FP+NS group. And paresthesia typically occurred 40–60 s after administration, accompanied by postadministration increases in plasma phosphate levels in both groups.

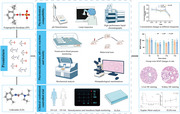

## 1 Introduction

Fospropofol disodium (fospropofol), a water‐soluble prodrug of propofol, has been developed to address the limitations of traditional propofol. Similar to propofol, fospropofol exerts sedative and anesthetic effects by facilitating chloride influx through γ‐aminobutyric acid (GABA) receptors and inhibiting N‐methyl‐d‐aspartate receptor‐mediated calcium influx. Previous studies have indicated that fospropofol could lead to significant paresthesia, with patients reporting sensations of pruritus, pinching, tingling, or burning after administration. These symptoms occurred predominantly in the perineal region, with an incidence rate of 62% [1, 2]. The development of paresthesia is thought to be related to the release of phosphate generated during fospropofol metabolism via alkaline phosphatases, a mechanism similar to that observed with other phosphate‐ester drugs, including dexamethasone [3, 4]. Currently, effective preventive strategies for this adverse event remain severely limited.

Lidocaine, an intermediate‐acting local anesthetic, acts as a sodium channel blocker and is widely used for both local anesthesia and systemic administration. Previous research has shown that lidocaine, when administrated in combination with propofol, effectively reduced injection pain by reversibly blocking peripheral nerve pathways through excitable membranes [5, 6, 7]. Clinical research has also indicated that lidocaine might decrease the incidence of perineal pruritus induced by dexamethasone [8]. Furthermore, lidocaine has displayed potential in managing refractory pruritus in patients with atopic dermatitis, chronic cholestatic liver diseases, or cutaneous T‐cell lymphoma [9, 10, 11, 12]. However, whether lidocaine could reduce the risk of fospropofol‐induced paresthesia remains uncertain.

To evaluate the safety and pharmacodynamic changes of premixed lidocaine with fospropofol, we conducted a series of preclinical experimental studies, including both in vitro and in vivo experiments, before initiating the randomized controlled (RCT) trial. Subsequently, an RCT was performed to explore the clinical effect of premixed lidocaine with fospropofol on the incidence of fospropofol‐induced paresthesia during induction.

## 2 Results

### 2.1 Preclinical Experimental Study

#### 2.1.1 Physicochemical Compatibility

**FIGURE 1:**
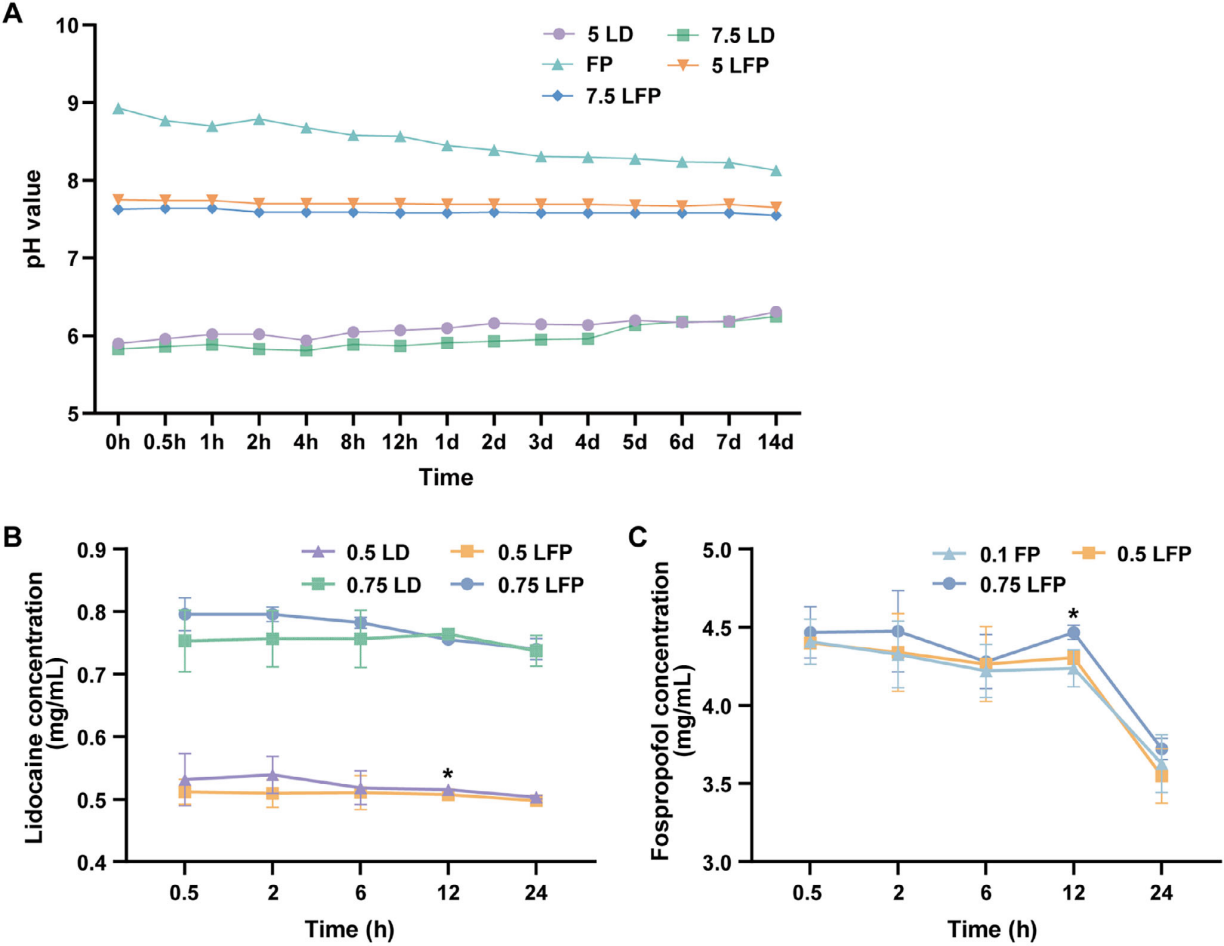
Trends in the pH value and alterations in drug concentration within the mixture of lidocaine and fospropofol. (A) Changes of pH value in mixed solution within 14 days (*n* = 3). (B) The result of high‐performance liquid chromatography (HPLC) about the concentration of lidocaine in mixed solution (*n* = 3). (C) The result of HPLC about the concentration of fospropofol in mixed solution (*n* = 3). 5 LD 5 mg/mL lidocaine, 7.5 LD 7.5 mg/mL lidocaine, FP 50 mg/mL fospropofol, 5 LFP 5 mg/mL lidocaine plus 50 mg/mL fospropofol, 7.5 LFP 7.5 mg/mL lidocaine plus 50 mg/mL fospropofol, 0.5 LD 0.5 mg/mL lidocaine, 0.75 LD 0.75 mg/mL lidocaine, 0.5 LFP 0.5 mg/mL lidocaine plus 5 mg/mL fospropofol, 0.75 LFP 0.75 mg/mL lidocaine plus 5 mg/mL fospropofol, and 0.1 FP 5 mg/mL fospropofol. Data are shown as mean ± sd. Comparing lidocaine concentrations, 0.5 LD versus 0.5 LFP, *p < 0.05; Comparing fospropofol concentrations, 0.5 LFP versus 0.75 LFP, *p < 0.05.

Measurement of the pH values across five mixed drug solutions at different concentrations and time points revealed that the pH values of the 5 LFP group and 7.5 LFP group were closer to the normal physiological pH range of the human body (pH 7.35–7.45) and remained more stable within 14 days compared with those of 5 LD, 7.5 LD, and FP group (Table S7 and Figure 1A). These findings indicated that the pH value of the mixture of fospropofol and lidocaine was suitable and stable for human physiology.

The outcome of lamp inspection experiment demonstrated that all mixed solutions remained clear, colorless, and free of crystallization or precipitation throughout the 14 days following preparation.

The results of high‐performance liquid chromatography (HPLC) were presented in Figure 1B,C. No difference in the concentration of lidocaine was observed between the 0.5 LD group and 0.5 LFP group within 6 h after solution preparation in vitro. The same situation also occurred in 0.75 LD group and 0.75 LFP group (Figure 1B). There was no difference in the concentration of fospropofol among 0.1 FP group, 0.5 LFP group, and 0.75 LFP group within 6 h after solution preparation in vitro (Figure 1C), suggesting that lidocaine did not affect the concentration of fospropofol within 6 h in mixed solution in vitro.

#### 2.1.2 Drug Toxicity Study

**FIGURE 2:**
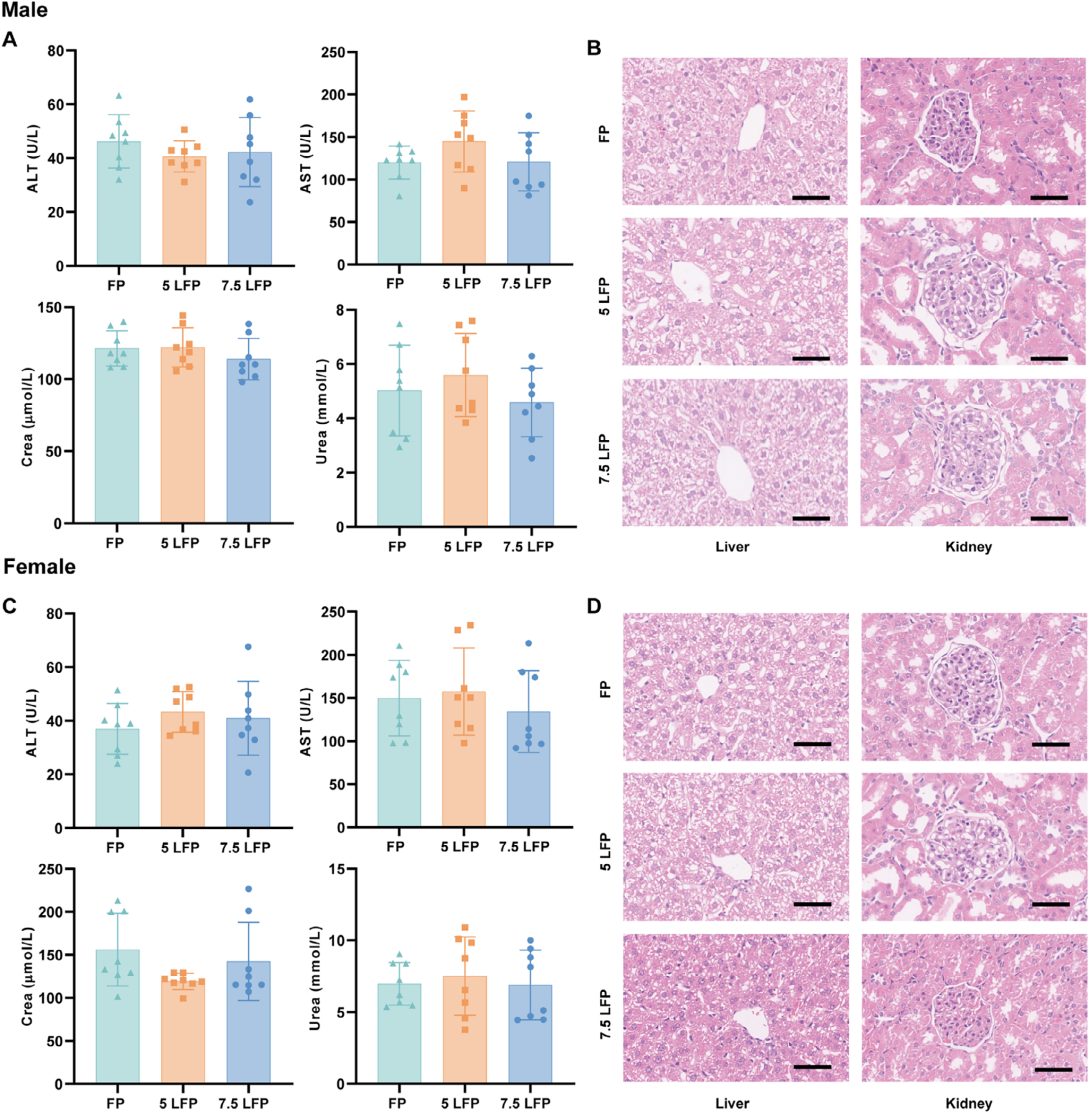
The function assessment and histopathological manifestations of liver and kidney in male or female rats at 7 days postadministration in different dose groups. (A) The concentration changes of ALT, AST, creatinine, and urea in male rats, and no significant differences in liver and kidney function were detected among the three groups (*n* = 8). (B) The representative HE staining images of liver and kidney in male rats, and the results showed that the overall structure and cellular morphology of each group were comparable, and no histopathological changes caused by research drugs were observed. The hepatocytes were arranged normally, with no necrosis or inflammation, and the structure of renal glomeruli and tubules remained intact. original magnification ×100, scale bars 50 µm (*n* = 8). (C) The concentration changes of ALT, AST, creatinine, and urea in female rats, and no significant differences in liver and kidney function were detected among the three groups (*n* = 8). (D) The representative HE staining images of liver and kidney in female rats, and the results also showed that the overall structure and cellular morphology of each group were comparable, and no histopathological changes caused by research drugs were observed. The hepatocytes were arranged normally, with no necrosis or inflammation, and the structure of renal glomeruli and tubules remained intact. original magnification ×100, scale bars 50 µm (*n* = 8). FP, 50 mg/mL fospropofol; 5 LFP, 5 mg/mL lidocaine plus 50 mg/mL fospropofol; 7.5 LFP, 7.5 mg/mL lidocaine plus 50 mg/mL fospropofol; ALT, alanine aminotransferase; AST, aspartate aminotransferase; Crea, creatinine; HE, hematoxylin–eosin. Data are shown as mean ± sd.

At 7 days after administration, no significant differences were observed in plasma alanine aminotransferase (ALT), aspartate aminotransferase (AST), creatinine, and urea concentrations among the three groups for male rats, and similarly, no significant differences were observed among the three groups for female rats (Figure 2A,C). Hematoxylin–eosin staining further revealed no pathological changes in either the liver and kidney cells of male and female rats after 7 days of administration in the three groups. The pathological findings of different organs were shown in Figure 2B,D.

#### 2.1.3 Pharmacodynamics and Adverse Reaction Research in Rats

The sedative effects of the drugs were analyzed based on the righting reflex data from the rats. Compared with the FP group, both the 5 LFP and 7.5 LFP groups exhibited a significantly shorter time to sedation onset and a longer duration of sedation, while no significant difference was shown in the recovery time among all groups (Table 1). These results demonstrated that the mixture markedly accelerates sedation onset and prolong the duration of sedation.

**TABLE 1:** Comparison of sedative, analgesic, and adverse reactions outcomes among three groups in animal study. Data are presented as *n* (%), or mean [standard deviation].

Variables	FP (*n* = 11)	5 LFP (*n* = 11)	7.5 LFP (*n* = 11)	*p* Value
Sedation onset time (s)	133.2 [14.9]	97.0 [13.3]^a^	68.6 [26.3]^a^, ^b^	<0.001
Duration of sedation (min)	25.9 [4.3]	31.6 [7.2]^a^	31.8 [4.3]^a^	0.024
Recovery time (min)	10.1 [2.6]	7.8 [2.2]	8.0 [2.6]	0.065
Incidence of pinch reflex disappearance (*n*, %)	1 (9.1%)	3 (27.3%)	10 (90.9%)^a^, ^b^	<0.001
Incidence of scratching behavior (*n*, %)	0	0	0	/
limb twitching (*n*, %)	2 (18.2%)	1 (9.1%)	1 (9.1%)	>0.990

Abbreviations: 7.5 LFP group, 7.5 mg/mL lidocaine plus 50 mg/mL fospropofol group; FP group, 50 mg/mL fospropofol group; 5 LFP group, 5 mg/mL lidocaine plus 50 mg/mL fospropofol group.

^a^ Compared with FP group, *p* < 0.05.

^b^ Compared with 5 LFP group, *p* < 0.05.

The incidence of pinch reflex disappearance reflected the analgesic effect of study drugs. In comparison with the FP group and 5 LFP group, the 7.5 LFP group demonstrated a significantly higher incidence of pinch reflex disappearance (Table 1), suggesting that the mixture could notably enhance the analgesic effect.

**FIGURE 3:**
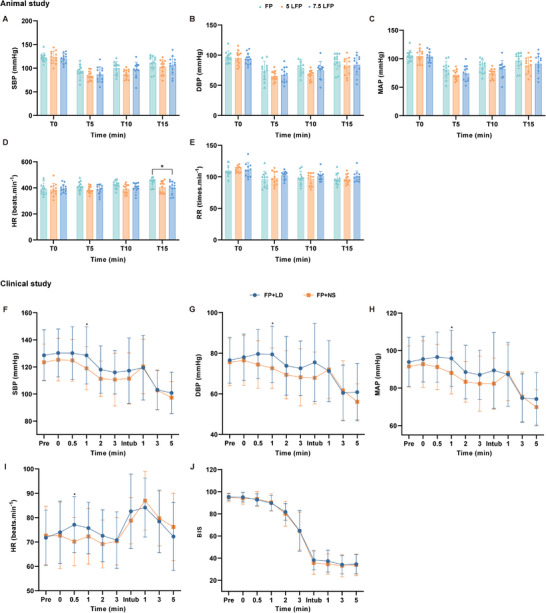
The influence of the experimental drug on the vital signs and anesthesia depth in rats and patients. The change trends in hemodynamics and respiratory rate between groups in animal study (A–E). (A) The change trend of SBP (*n* = 12). (B) The change trend of DBP (*n* = 12). (C) The change trend of MAP (*n* = 12). (D) The change trend of HR (*n* = 12). (E) The change trend of RR (*n* = 11). The change trends in hemodynamics and BIS between FP+LD and FP+NS group in clinical study (F–J). (F) The change trend of SBP. (G) The change trend of DBP. (H) The change trend of MAP. (I) The change trend of HR. (J) The change trend of BIS. FP, 50 mg/mL fospropofol; 5 LFP, 5 mg/mL lidocaine plus 50 mg/mL fospropofol; 7.5 LFP, 7.5 mg/mL lidocaine plus 50 mg/mL fospropofol; SBP, systolic blood pressure; DBP, diastolic blood pressure; MAP, mean arterial pressure; HR, heart rate; RR, respiratory rate; BIS, bispectral index. Data are shown as mean ± sd. In animal study, FP versus 7.5 LFP, *p < 0.05. In clinical study, FP+LD versus FP+NS, *p < 0.05.

No significant differences were observed in SBP, diastolic blood pressure (DBP), mean arterial pressure (MAP), or respiratory rate among three groups (Figure 3A–C,E). Although heart rate (HR) differed significantly statistically between the FP and 7.5 LFP groups at T15 point, the magnitude of this difference is unlikely to be clinically meaningful (Figure 3D). Hence, the mixture did not notably increase the risks related to respiratory or hemodynamic adverse events.

No scratching behavior was observed in any group during the induction period (Table 1), and there were no significant differences in adverse reactions, which mainly included limb twitching (Table 1).

### 2.2 Clinical RCT

Based on the findings of preclinical studies, the clinical study was deemed safe to proceed in order to evaluate the effects of premixed lidocaine with fospropofol on paresthesia induced by fospropofol.

**FIGURE 4:**
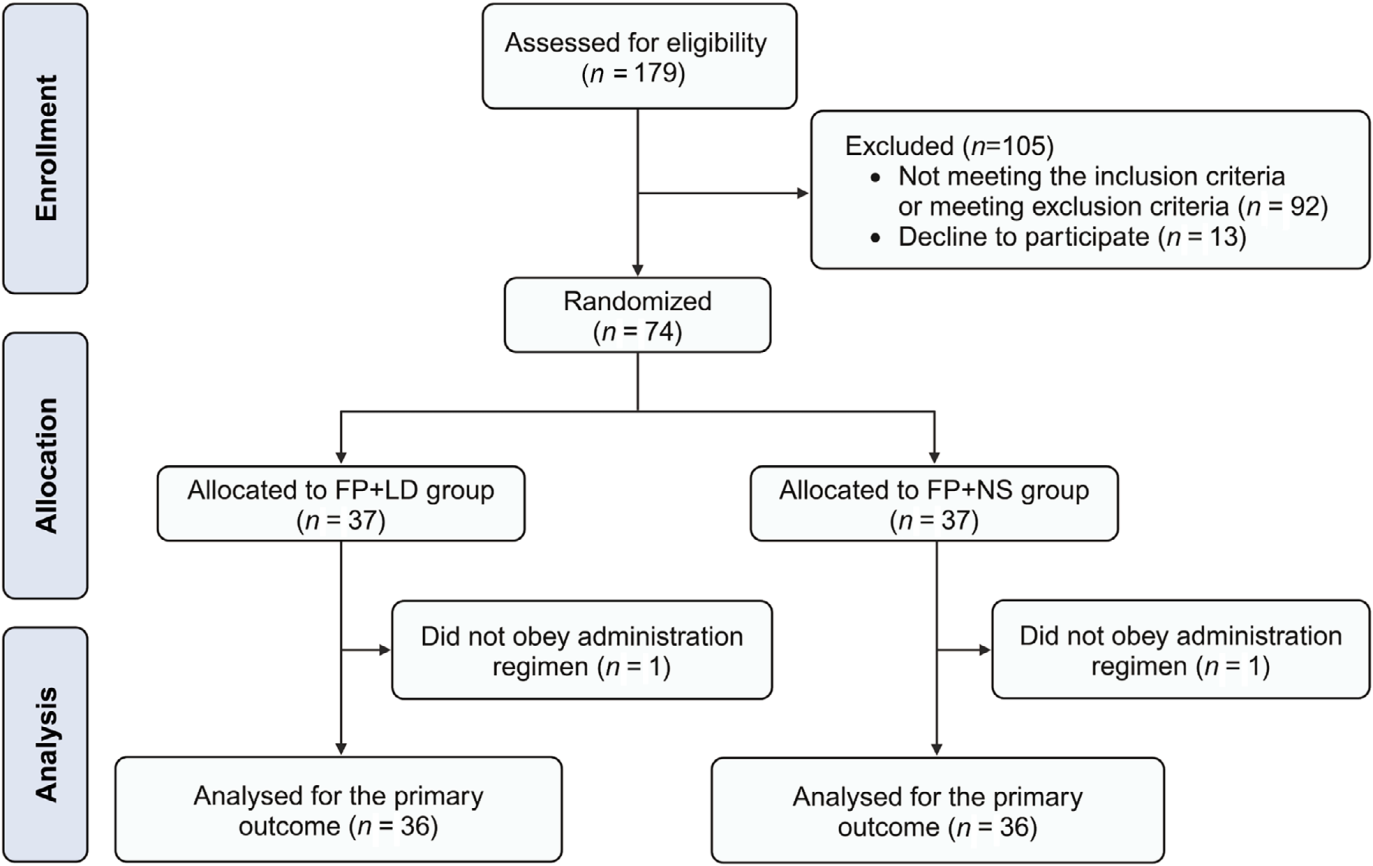
The CONSORT flow diagram of the randomized clinical controlled trial.

In the clinical study, a total of 179 patients undergoing elective surgery were screened between May 2023 and September 2023. Subsequently, 74 patients were included and randomized into either the FP+LD group (*n* = 37) or the FP+NS group (*n* = 37). One patient from each group was excluded due to the violation of the study protocol, which affecting the primary outcome evaluation. Therefore, a total of 36 patients in each group were included in the primary outcome analysis. Additionally, two patients in the FP+NS group were excluded from the secondary outcome analysis because anesthesia induction with fospropofol could not be completed. No patients were lost to follow‐up. The flow diagram was presented in Figure 4.

#### 2.2.1 Baseline Characteristics and Intraoperative Data

The baseline characteristics and intraoperative data of all participants were presented in Table 2. All baseline variables were balanced between the FP+LD group and the FP+NS group.

**TABLE 2:** Baseline and surgical characteristics in the two groups. Data are presented as *n* (%), median (interquartile range), or mean [standard deviation].

Characteristics	FP+LD group (*n* = 36)	FP+NS group (*n* = 36)
Age, years	42.5 [11.2]	39.1 [10.1]
Sex		
Female	27 (75.0%)	28 (77.8%)
Male	9 (25.0%)	8 (22.2%)
Weight, kg	61.3 [9.1]	58.7 [8.5]
BMI, kg/m^2^	23.3 [2.6]	22.5 [2.7]
ASA physical status		
I	2 (5.6%)	5 (13.9%)
II	34 (94.4%)	31 (86.1%)
Educational level		
Elementary education or less	1 (2.8%)	1 (2.8%)
Middle or high school education	10 (27.8%)	7 (19.4%)
College‐level education or higher	25 (69.4%)	28 (77.8%)
Medication		
None	31 (86.2%)	33 (91.7%)
Angiotensin receptor blocker	1 (2.8%)	0 (0)
Calcium channel blocker	2 (5.6%)	1 (2.8%)
Hormonal drugs	2 (5.6%)	2 (5.6%)
Baseline hemodynamic variable		
Systolic blood pressure, mmHg	120 [16]	117 [14]
Diastolic blood pressure, mmHg	78 [12]	78 [10]
Heart rate, beats/min	78 [13]	83 [11]
Blood ALP (IU/L)	82.1 [37.4]	73.0 [18.7]
Induction medications		
Sufentanil, mg	18.4 [2.7]	17.8 [2.5]
Rocuronium, mg	36.8 [5.5]	35.6 [5.0]
Fospropofol disodium, mg	629.6 [95.2]	626.4 [106.0]
Surgical procedures		
LC	30 (83.3%)	29 (80.6%)
Intestinal surgery	1 (2.8%)	1 (2.8%)
Thyroid or breast surgery	3 (8.3%)	6 (16.7%)
Lower extremity varicose vein surgery	2 (5.6%)	0 (0)

Abbreviations: ALP, alkaline phosphatase; ASA, American Society of Anesthesiologists Physical Status; BMI, body mass index; FP, fospropofol; LC, laparoscopic cholecystectomy; LD, lidocaine; NS, normal saline.

#### 2.2.2 Paresthesia‐Related Outcomes

The incidence of paresthesia did not differ significantly between the FP+LD group and FP+NS group (*p > *0.990). Lidocaine also did not significantly prolong the initial onset time and duration of paresthesia induced by fospropofol (*p *= 0.862 and *p *= 0.838, respectively) (Table 3 and Figure S1). Tingling was the most frequently reported paresthesia, occurred in 18 patients (50%) in the FP+LD group and 19 patients (52.8%) in the FP+NS group (Table 3). In both groups, paresthesia predominantly affected the perineal region. Notably, the absolute proportion of paresthesia occurring in the whole body in the FP+NS group (27.8%) was higher than that in the FP+LD group (11.1%), although the overall difference in the bodily location of paresthesia was not statistically significant (*p *= 0.202) (Table 3). Furthermore, lidocaine did not reduce itching intensity, as indicated by Verbal Rating Score (VRS) of 0 (0–2) in both groups (*p *= 0.967) (Table 3). Additionally, the incidence of injection pain was negligible, with no patients in the FP+LD group and only one patient in the FP+NS group experiencing this adverse sensation (Table S8).

**TABLE 3:** Comparison of paresthesia‐related outcomes and induction success rate between two groups in clinical study. Data are presented as *n* (%), median (interquartile range), or mean [standard deviation].

Outcomes	FP+LD group (*n* = 36)	FP+NS group (*n* = 36)	*p* Value
Incidence of paresthesia	30 (83.3%)	30 (83.3%)	>0.990
Initial onset time of paresthesia, s	45 (30–45)	45 (30–45)	0.862
Duration of paresthesia, s	70 (45–101.25)	72.5 (45–90)	0.838
Type of paresthesia			>0.990
None	6 (16.7%)	6 (16.7%)	
Pruritus	3 (8.3%)	3 (8.3%)	
Tingling sensation	18 (50%)	19 (52.8%)	
Others	9 (25.0%)	8 (22.2%)	
Bodily location of paresthesia			0.202
None	6 (16.7%)	6 (16.7%)	
Perineum or perineum plus other sites	24 (66.7%)	17 (47.2%)	
Whole body	4 (11.1%)	10 (27.8%)	
Lower body	1 (2.8%)	3 (8.3%)	
Other individual location	1 (2.8%)	0 (0)	
Pruritus score (median, range)	0 (0–2)	0 (0–2)	0.967
Recall incidence of paresthesia	7 (19.4%)	11 (30.6%)	0.276
Success rate of induction	36 (100%)	34 (94.4%)	0.493

Abbreviations: FP, fospropofol; LD, lidocaine; MOAA/S, Modified Observer's Assessment of Alertness/Sedation Scale; NS, normal saline; PACU, postanesthesia care unit.

#### 2.2.3 Sedation Outcomes

No statistically significant differences were observed in sedation outcomes between the two groups, including the success rate of induction, time to reach a Modified Observer's Assessment of Alertness/Sedation Scale (MOAA/S) ≤ 1, time to loss of eyelash reflex, time to intubation, and time to initiation of anesthesia maintenance (Tables 3 and S8). The alteration trend of bispectral index (BIS) was consistent in both groups, with no notable variance at each designated time point (Figure 3J).

#### 2.2.4 Hemodynamic Outcomes

A total of 13 out of 35 patients in the FP+LD group and 12 out of 33 patients in the FP+NS group experienced induction hypotension, with no statistically significant difference (*p *= 0.947). Patients in the FP+LD group had significantly higher SBP, DBP, and MAP at 1 min after administration (*p *< 0.05), compared with FP+NS group (Figure 3F–H). However, there were no differences in SBP, DBP, and MAP between the two groups at other time points after administration (Figure 3F–H). At the point of 0.5 min after administration, HR showed statistically significant difference (*p *< 0.05), but no differences were detected at other time points (Figure 3I).

#### 2.2.5 Satisfactory Assessment and Other Complications

Two patients in the FP+NS group rated their assessment results as neutral‐satisfied, while all patients in the FP+LD group reported overall satisfaction with their experience of induction. Three anesthesiologists expressed dissatisfaction with the induction in FP+NS group, while none in the FP+LD group, with no statistically significant difference between the two groups (Table S9). One patient in the FP+LD group experienced transient tinnitus, and three patients in the FP+NS group had complications such as overexcitement, transient flushing in the upper chest and neck, and nausea (Table S8).

#### 2.2.6 Plasma‐Test Outcomes

**FIGURE 5:**
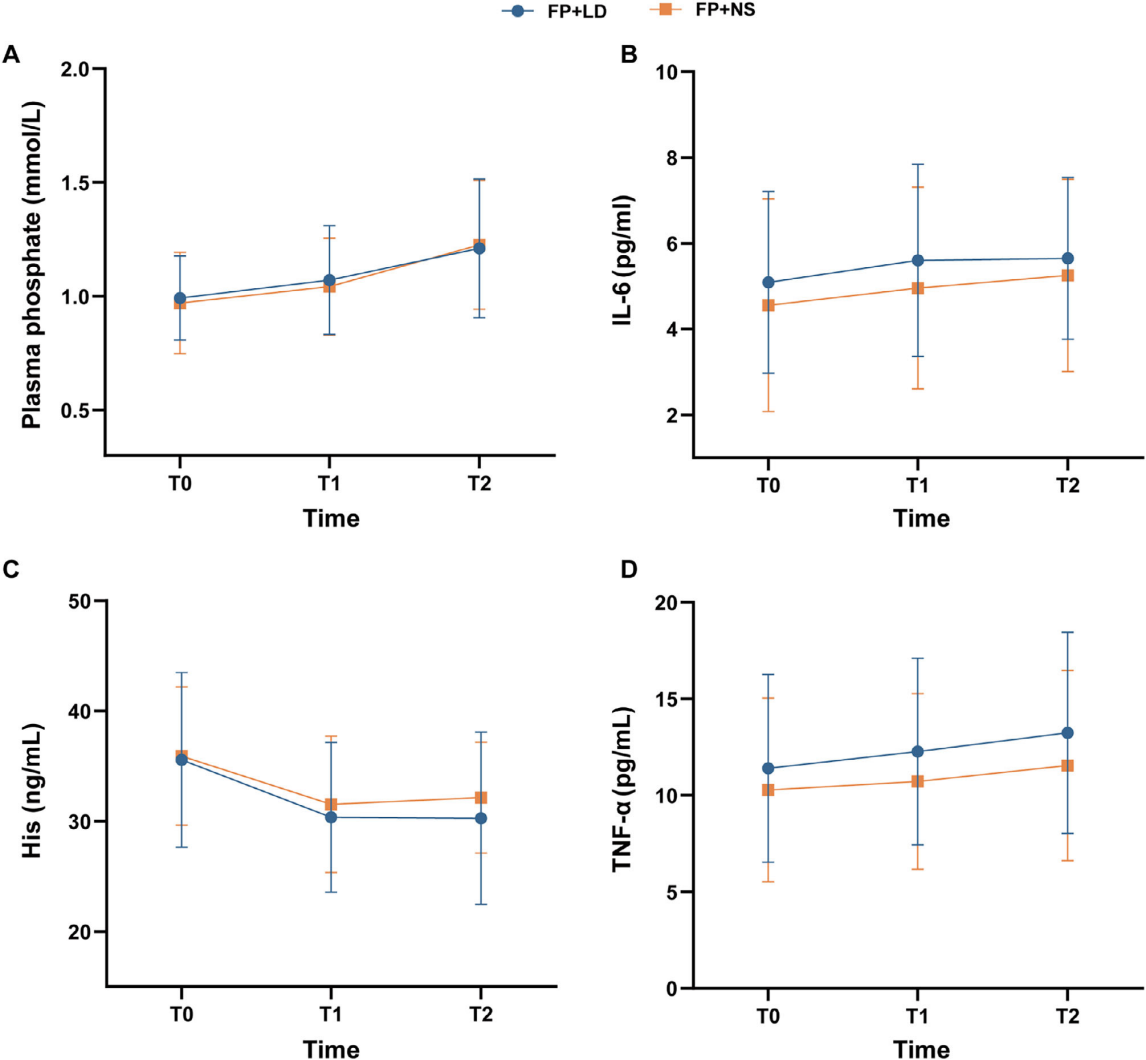
Concentration changes of plasma phosphate and inflammatory cytokine between FP+LD and FP+NS group at three time points in the clinical study. (A) The concentration of plasma phosphate. (B) The concentration of IL‐6. (C) The concentration of His. (D) The concentration of TNF‐α. IL‐6, interleukin‐6; TNF‐α, tumor necrosis factor‐alpha; His, histamine. Data are shown as mean ± sd.

Due to unsuccessful blood sampling in some patients, plasma analyses were completed for a total of 63 participants. No significant differences were observed between the FP+LD and FP+NS group in any of the laboratory indicators (Figure 5). However, plasma phosphate concentration was higher at T1 than T0 in those at both groups (*p *< 0.05) (Figure 5A). Additionally, the concentration of histamine (His) was lower at T1 compared with T0 in both groups (*p *< 0.05) (Figure 5C).

## 3 Discussion

In the preclinical study, the mixture of lidocaine and fospropofol demonstrated stable physicochemical compatibility with no evidence of drug toxicity. More importantly, in the rat model, the mixture of lidocaine and fospropofol could notably reduce the sedation onset time and prolong the duration of sedation, without changing hemodynamics or increasing the risk of adverse reactions. However, the findings from clinical studies indicated that intravenous lidocaine did not significantly alleviate fospropofol‐induced paresthesia. The clinical study also found that the paresthesia induced by fospropofol primarily occurred 40–60 s after administration, and plasma phosphate levels may contribute to its development.

Previous clinical studies have demonstrated that intravenous lidocaine at a dose of 1.5 mg/kg could effectively reduce dexamethasone‐induced perineal pruritus compared with placebo [8]. However, our clinical study found that the use of 1.5 mg/kg lidocaine did not reduce the incidence of paresthesia caused by fospropofol. This discrepancy suggested a potential difference in the mechanisms underlying paresthesia induced by fospropofol and dexamethasone, which might account for variations in the therapeutic efficacy of lidocaine. Notably, although the incidence of paresthesia was not statistically different between groups, we observed a lower absolute proportion of whole‐body paresthesia in the FP+LD group (11.1%) compared with the FP+NS group (27.8%), indicating that lidocaine might have some effect on limiting whole‐body paresthesia occurrence, and future studies with larger sample sizes are warranted to further investigate this potential advantage.

Several studies have reported associations between paresthesia and the release of inflammatory factors and blood substances, including His, interleukin (IL)‐6, tumor necrosis factor‐alpha (TNF‐α), and blood phosphate [3, 13, 14, 15, 16]. His is known to be released from mast cells when tissues during inflammation or allergen exposure [17, 18]. Previous literature has reported that the levels of His and IL‐6 are elevated in the serum of atopic dermatitis rat [19]. Elevated IL‐6 mRNA levels were also demonstrated in the transcriptome of skin tissues of patients with atopic dermatitis and prurigo nodularis [20]. Furthermore, increased mRNA levels of TNF‐α were also detected in the lesional skin of a pruritic trichophytin contact dermatitis mouse [21]. Moreover, uremic patients frequently have skin pruritus and abnormal phosphate metabolism. Clinical studies have shown that uremic pruritus may be due to the increased content of divalent ions in the skin, resulting in microprecipitation of calcium or magnesium phosphate, and the resolution of pruritus following UVB treatment was associated with a reduction of skin phosphorus [22, 23]. Higher residual estimated glomerular filtration rate and lower serum phosphate level were related with lower burden of chronic kidney disease‐associated pruritus in dialysis patients [24]. To explore whether lidocaine modulated these factors, we evaluated the levels of these substances in the patients’ plasma. No statistically significant differences were identified in these plasma substances between the two groups, suggesting that lidocaine had no discernible effect on these factors. Nonetheless, the involvement of these factors in fospropofol‐induced paresthesia remains plausible. As phosphorylation modification, a common approach for drug improvement in the field of pharmacy, becomes increasingly prevalent, pruritus is gradually found to be one of the common side effects and the mechanism is unknown, for example, hydrocortisone sodium phosphate [25].

In our study, we also noted elevated plasma phosphate concentrations at T1 point in both groups compared with T0 point. Interestingly, recent studies have discovered that the potential target of phosphate modified compounds is Mas‐related G protein‐coupled receptor X4 (MRGPRX4), a primate‐specific, sensory neuron receptor previously implicated in itch [26]. This result prompted us to postulate that plasma phosphate might play a critical role in fospropofol‐induced paresthesia, which was anticipated to be further investigated in future research.

Paresthesia represents an adverse subjective experience reported by conscious patients during induction of anesthesia, a critical period in which patients transition from consciousness to sedation. Therefore, the paresthesia induced by fospropofol during this period has a specific temporal characteristic. In this study, the posthoc time‐to‐event analysis revealed that paresthesia induced by fospropofol primarily occurred 40–60 s after administration (Figure S1). Therefore, further research is requisite to explore more effective methods for anesthesia induction that could facilitate a more rapid transition into a sedative state for patients.

In our preclinical animal study, the results indicated that combining lidocaine with fospropofol might enhance sedative performance. Compared with fospropofol alone, the mixture showed a faster onset, longer duration of sedation, and a noticeable analgesic effect. These effects may be attributed to lidocaine‐mediated sodium channel inhibition and reduced neuronal excitability, potentially producing a synergistic enhancement of the sedative response [27]. Additionally, lidocaine has been reported to modulate GABAergic signaling and other inhibitory pathways, potentially enhancing the sedative efficacy of GABA‐related anesthetics [28]. Although such synergy was not replicated in our clinical trial, the absolute values showed a numerically shorter onset time in the FP+LD group comparing with the FP+NS group, and the trend was consistent with the preclinical findings. This suggests that lidocaine may similarly potentiate the sedative effect of fospropofol in humans. However, because this variable was a secondary outcome, the clinical trial might not be powered to detect modest between‐group differences, which may explain the absence of statistical significance despite the observed numerical trend. Future studies with larger sample sizes focused specifically on sedation‐related endpoints are warranted to confirm the clinical relevance of this potential synergistic interaction.

The clinical study demonstrated that patients in the FP+LD group had significantly higher blood pressure at 1 min after administration and showed smaller overall hemodynamic fluctuations, compared with FP+NS group. The finding was consistent with previous study results, indicating that intravenous lidocaine could reduce hemodynamic changes during perioperative period [29]. And premixed lidocaine with fospropofol may therefore contribute to mitigating fospropofol‐induced circulatory depression, which should be further investigated through large‐scale studies.

This study has several limitations. First, since fospropofol induces rapid loss of behavioral control in rats, scratching behavior could not be reliably assessed, precluding evaluation of pruritus‐related outcomes in this animal study. Second, the clinical study was a single‐center RCT, which might have affected the generalizability of the findings. Third, the subjective nature of assessment methods for paresthesia‐related outcomes and potential bias from frequent patient questioning could artificially inflate reported incidence rates and mask lidocaine's true effects. Finally, due to limited clinical blood samples and detectable indicators, not all paresthesia‐related biochemical markers could be comprehensively assessed based on literature reports.

## 4 Conclusion

In summary, premixed lidocaine with fospropofol did not reduce the incidence of fospropofol‐induced paresthesia. However, our findings indicated that the duration of paresthesia was transient and paresthesia very mild in severity. Moreover, paresthesia induced by fospropofol primarily occurs 40–60 s after administration, and plasma phosphate might play a role in its occurrence.

## 5 Materials and methods

### 5.1 Preclinical Experimental Study

#### 5.1.1 Experimental Animals and Medications

Six‐ to eight‐week‐old male and female Sprague–Dawley (SD) rats weighing 280–300 g were obtained from Chengdu Dashuo Experimental Animal Co., Ltd. (Sichuan, China). Animals were housed in groups of three to five per cage and were acclimated for 7 days before the experiment under controlled environment at 20–25°C with 38–41% humidity and a 12‐h light/dark cycle, with supply of standard food and water. This research was granted clearance by the Animal Care and Use Committee of our institution (Approval number: 20230330002). All experimental and animal handling procedures were conducted strictly according to the Guide for the Care and Use of Laboratory Animals published by the National Institutes of Health.

Fospropofol, a novel intravenous anesthetic employed in this study, was provided by Yichang Humanwell Pharmaceutical Co., Ltd. (Hubei, China). This compound was approved as a Class 1.1 natural new drug by China's National Medical Products Administration (NMPA) in 2021. Lidocaine hydrochloride (2%) was purchased from Shiyao Yinhu Pharmaceutical Co., Ltd. (Shanxi, China). This study was reported based on the ARRIVE guidelines [30].

#### 5.1.2 pH Determination and Lamp Inspection

Samples of drug solutions were examined using pH meter (Sartorius PB‐30L) and visually inspected under a lamp immediately after preparation of the drug (0 h) and at 0.5 h, 1 h, 2 h, 4 h, 8 h, 12 h, 1 day, 2 days, 3 days, 4 days, 5 days, 6 days, 7 days, and 14 days [31]. The study groups and methods of drug preparation were shown in Table S1.

#### 5.1.3 HPLC Test

The mobile phase consisted of acetonitrile and 0.1% trifluoroacetic acid at a flow rate of 1.0 mL/min, with the column temperature set to 25°C. Lidocaine was detected at a wavelength of 263 nm using a UV spectrophotometer, with an acquisition time of 7 min [32]. Fospropofol was detected by a fluorescence spectrophotometer with an acquisition time of 12 min [33]. The sample load was 10 µL. As the initial concentrations of lidocaine and fospropofol were too high for direct HPLC detection, all samples were subsequently diluted 10‐fold while maintaining a constant concentration ratio between lidocaine and fospropofol. The methods of drug preparation for each group were shown in Table S2.

#### 5.1.4 Drug Toxicity Study

At 7 days after administration, venous blood samples were collected from rats. Plasma was separated by centrifugation at 3500 rpm for 10 min for liver and kidney function testing (Mindray BS‐120).

After euthanizing, the rats were perfused with cold phosphate‐buffered saline and paraformaldehyde (PFA) through heart. The liver and kidney were fixed with 4% PFA and embedded in paraffin. Slices of 3–4 µm thickness were stained with hematoxylin–eosin [34].

The SD rats were randomly assigned to three groups based on different drug treatments, as described in Table S3.

#### 5.1.5 Evaluation of Sedative and Analgesic, Respiratory and Hemodynamics, Itching, and Adverse Reactions

The sedative effect was evaluated using the righting reflex evaluation scale (Table S4) [35]. The analgesic effect was evaluated using the finger pinching reflex score (0 = no response; 1 = response) [36]. Baseline scores for both righting and pinching reflexes were recorded before administration. The righting reflex of rats was evaluated separately after administration, according to the criteria presented in Table S5. The evaluation method of the pinching reflex was as follows: The rat's right hind paw was gently but firmly pinched with hemostatic forceps to determine whether a leg‐withdrawal response occurred, and the absence of leg contraction indicated the disappearance of pain response. Baseline respiratory rates were measured before each dose by counting abdominal movements over 30 s. The blood pressure and HR were recorded at baseline and intervals of 5, 10, and 15 min after administration by a noninvasive measurement analysis system (Nanjing Karwin Biotechnology Co., Ltd, Nanjing, China). Pruritus was assessed by observing the rats’ scratching behavior during the period from administration to the disappearance of the righting reflex [37]. Any adverse reactions during the study would be recorded. The study groups were shown in Table S3.

#### 5.1.6 Statistical Analysis Methods

The experimental data were calculated using GraphPad Prism 9.5 and SPSS IBM Version 25.0 statistical data processing software, with results expressed as mean and standard deviation. ANOVA test was used for comparison between multiple mean groups, and the Tukey method for pairwise comparison. Analgesic rates were compared using the chi‐square test. *p *< 0.05 indicated that the difference was statistically significant.

### 5.2 Clinical Study

#### 5.2.1 Study Design

The RCT was approved by the Ethics Committee of our Hospital (Approval number: 2022‐1273), and informed consents were obtained from all patients prior to enrollment. This study was registered with the Chinese Clinical Trial Registry on May 11, 2023 (Clinical trial number: ChiCTR2300071330). The work of this RCT has been reported based on the Consolidated Standards of Reporting Trials (CONSORT) guideline [38].

#### 5.2.2 Inclusion and Exclusion Criteria

The study enrolled patients aged 18–65 years, with a body mass index (BMI) ranging from 18 to 30 kg/m^2^, classified as the American Society of Anesthesiologists classification of I–III, scheduled for elective noncardiothoracic and non‐neurological surgeries requiring endotracheal intubation under general anesthesia. Eligible participants had no history of psychiatric disorders, chronic pruritus, or paresthesia, and signed informed consent.

The exclusion criteria included as follows: (1) patients with contraindications to general anesthesia or a history of anesthesia‐related complications; (2) patients planned for additional anesthetic techniques, such as epidural, spinal anesthesia, or nerve block; (3) individuals with difficult airways or assessed as having difficult tracheal intubation (Modified Mallampati score of III or IV); (4) patients with history of neuropsychiatric disorders, including traumatic brain injury, intracranial hypertension, stroke, or a history of mental illness; (5) liver function insufficiency (ALT or AST ≥2.5 times the upper limit of normal, or total bilirubin ≥ 1.5 times the upper limit of normal); (6) renal function insufficiency (creatinine levels above the upper limit of normal, or undergoing dialysis treatment within 28 days prior to surgery); (7) uncontrolled diabetes, hypertension, allergic reactions to the trial medication or other anesthetic drugs; (8) patients who have participated in any clinical drug trials within 1 month before screening or are considered unsuitable for this trial due to other factors.

#### 5.2.3 Randomization, Allocation, and Blinding

Eligible patients were randomly assigned to either the FP+LD or FP+NS group by simple randomization in a 1:1 ratio based on a computer‐generated random number list. To guarantee allocation concealment, the assignment was enclosed in sequentially numbered opaque envelopes and opened by an independent researcher who had no involvement in anesthesia intervention or outcome assessment, only after obtaining written consent and upon the patient's arrival at the operating center. Study medications were prepared by an impartial researcher according to group allocation and administered using identical, and an unlabeled 20 mL syringe was used for administration. Since the solution of the drugs used in this research was colorless and transparent, the investigators, assessors, and patients remained fully blinded to group assignment throughout the study.

#### 5.2.4 Study Intervention

For the FP+LD group, during the preanesthesia medication preparation phase, 7.5 mL of 2% lidocaine was diluted with saline to a total volume of 20 mL. This solution was subsequently employed to dissolve 1000 mg of fospropofol (sterile, white, lyophilized powder; two vials of 500 mg fospropofol each) through thorough mixing, leading to a concentration of 50 mg/mL for fospropofol and 0.75% for lidocaine, respectively. For anesthesia induction, a dosage of fospropofol at 10 mg/kg was administered together with an intravenous bolus of lidocaine at a dose of 1.5 mg/kg. The lidocaine intervention dose (1.5 mg/kg) was specifically selected for this study, and the two agents were freshly mixed before induction.

For the FP+NS group, during the medication preparation, 1000 mg of fospropofol powder was dissolved in 20 mL of normal saline to achieve a concentration of 50 mg/mL. The dosage of fospropofol administered was 10 mg/kg in accordance with the anesthesia induction protocol.

#### 5.2.5 Rationale for the Dosage Selection of Lidocaine and Combination With Fospropofol

Intravenous lidocaine has been shown to reduce dexamethasone‐induced perineal pruritus during anesthesia induction, and combining lidocaine with propofol for anesthesia induction also alleviate propofol‐related injection pain [8, 39]. Previous studies have also demonstrated that a single dose of lidocaine exceeding 1.5 mg/kg exerts both peripheral and central effects while inhibiting the rate of propofol plasma concentration increase [40]. Based on this evidence, this study selected 1.5 mg/kg of lidocaine to premix with fospropofol, resulting in concentrations of 50 mg/mL for fospropofol and 0.75% for lidocaine.

#### 5.2.6 Anesthesia Protocol and Management of Adverse Reactions

In addition to the standard monitoring protocol, BIS monitoring was applied to guide anesthesia depth. After 3–5 min of preoxygenation with mask ventilation, general anesthesia was induced using fospropofol (Yichang Humanwell Pharmaceutical Co., Ltd., Hubei, China) at a dosage of 10 mg/kg. Once patients lost consciousness, subsequent administration included 2 mg midazolam, 0.3 µg/kg sufentanil, and 0.6 mg/kg rocuronium. Endotracheal intubation was performed following adequate muscle relaxation. Patients were ventilated with an oxygen concentration of 30–50% and a tidal volume of 6–8 mL/kg, with the respiratory rate adjusted to keep end‐tidal carbon dioxide levels between 35 and 45 mmHg. Anesthesia maintenance according to the institutional protocol involved the use of remifentanil in combination with other sedative medications to regulate the BIS within the range of 40–60, and the sedative maintenance drugs were given when BIS reached 55 postinduction. After surgery, patients were transferred to the postanesthesia care unit (PACU) and monitored according to the institutional PACU protocol.

Adverse reactions were continuously monitored throughout the whole study. Specifically, any adverse events experienced by the participants were promptly recorded and assessed by the study investigators. All adverse reactions were classified according to severity and type, and appropriate clinical management was provided when necessary. In addition, an independent safety monitoring committee regularly reviewed all adverse events to ensure patient safety.

#### 5.2.7 Blood Sample Collection and Testing

Blood samples were collected by a researcher who was not involved in the anesthesia intervention and outcome assessment, using precooled ethylene diamine tetraacetic acid anticoagulant tubes. Sampling time points were defined as follows: before drug administration (T0), after the patient lost consciousness without other induction drugs (T1), and when the conditions for tracheal intubation were met but intubation had not yet been carried out (T2). Immediately after collection, blood samples were centrifuged to obtain plasma, which was then stored in an ultra‐low‐temperature freezer at −80°C for subsequent laboratory analyses using enzyme‐linked immunosorbent assay and the phosphomolybdate method.

#### 5.2.8 Study Outcomes

The primary outcome was the incidence of paresthesia (such as itching, tingling sensation) from the initiation of using study drugs to loss of consciousness.

The secondary outcomes included: (1) other paresthesia‐related outcomes; (2) VRS for itching (0 = no itch; 1 = low itch; 2 = moderate itch; 3 = severe itch) from drug administration to loss of consciousness; (3) incidence of injection pain after administration; (4) indicators related to anesthesia induction, including the MOAA/S scale (Table S6), success rate of anesthesia induction (MOAA/S ≤ 1), time of eyelash reflex disappears, and time to intubation; (5) incidence of hypotension (systolic blood pressure (SBP) < 90 mmHg) and bradycardia (HR < 50 beats/min) during induction; (6) changes in blood pressure, HR, and BIS during induction (premedication, immediately after administration, 0.5, 1, 2, and 3 min postadministration, at intubation, 1, 3, and 5 min postintubation); (7) start time of maintenance anesthesia drugs; (8) recovery period related outcomes, including satisfaction assessment and recall incidence of paresthesia; (9) incidence of other adverse events.

Plasma‐test outcomes encompassed measurements of the following: plasma phosphate, His, IL‐6, and TNF‐α. Given the purpose of this study and the focus on the anesthesia induction period, the secondary outcome, vital signs during the anesthesia maintenance period, was not included in the final analysis.

#### 5.2.9 Data Collection

A well‐trained researcher, blinded to study group allocation, collected all perioperative data. Paresthesia was assessed at 15‐s intervals from after the administration of the study drug until the loss of consciousness through the following methods: (1) initially asking patients about any discomfort; (2) if the patients reported discomfort, we would further assess whether the discomfort was due to paresthesia. And the paresthesia was also assessed according to the patients’ behavior (such as scratching). The VRS was utilized to evaluate itching scores when it occurred. Vital signs and sedation‐related indicators were recorded at predetermined time points. Additionally, patients’ and anesthesiologists’ satisfaction levels were documented, and the satisfaction was assessed using a five‐point scale, including very satisfied, satisfied, neutral, dissatisfied, and very dissatisfied. And the patients’ satisfaction was assessed after regaining full consciousness in the PACU.

#### 5.2.10 Statistical Analysis

The statistical analysis of the primary outcome was conducted using the modified intention‐to‐treat method, excluding participants who did not obey the study protocol, resulting in difficulties in evaluating the primary outcome measure. The normality of continuous data was assessed using the Shapiro–Wilk test. Descriptive analysis for normally distributed variables utilized mean and standard deviation, while median and interquartile range or range were used for non‐normally distributed data. Dichotomous data were presented as frequency (percentage). Furthermore, a two‐sample *t*‐test was employed to compare mean differences between groups for normally distributed data, whereas the Mann–Whitney *U*‐test was used for non‐normally distributed data. A repeated measures ANOVA test was conducted to assess differences over time in hemodynamics, BIS, and plasma substance variables. *χ* ^2^ or Fisher's exact tests were utilized for dichotomous data. A posthoc Kaplan–Meier analysis of paresthesia was performed. All analysis was two‐tailed, with a significance level set at *p *< 0.05. Statistical analysis was performed using SPSS IBM version 25.0, GraphPad Prism 9.5 and Adobe Illustrator 2024 were employed for figure design.

#### 5.2.11 Sample Size Calculation

As there is currently no direct evidence regarding the effect of lidocaine on fospropofol‐induced paresthesia, the expected treatment effect was estimated based on previous studies. Lidocaine has been reported to reduce the relative risk of propofol injection pain by approximately 60% and dexamethasone sodium phosphateinduced pruritus by approximately 77.5% [8, 39]. Based on the above findings and difference between these conditions, we conservatively assumed that lidocaine could reduce the relative risk of fospropofol‐induced paresthesia by approximately 60%. And previous research has reported that the incidence of fospropofol‐induced paresthesia was 62% [2]. The sample size for the study was determined using the test for the two independent proportions model in PASS version 15 software. Assuming an alpha level of 0.05 (two‐sided), a beta level of 0.1, and a dropout rate of 10%, a total sample size of 37 patients per group was calculated to be necessary. Finally, a total of 74 patients were included in the study.

## Author Contributions

Study design: BJ, JL, and CC. Coordination: PL, JL, and CC. Patient recruitment: BJ and CC. Data collection: XLX, YC, DQZ, QY, YL, XHS, MQX, TL, HX, and XJT. Data interpretation: BJ and CC. Writing of the first draft of the manuscript: BJ, XLX, CYY, JQX, and LYD. Critical revision of the manuscript: CC, JL, and XQL. The manuscript has been read and approved by all the authors.

## Funding Information

The study was supported by Yichang Humanwell Pharmaceutical Co., Ltd., Hubei, China. The funding organization had no role in the study design, data collection, analysis, interpretation of data, or writing of the manuscript.

## Ethics Statement

The written informed consent had got from all patients to participate in the study. This trial has received approval from the ethics committee of West China Hospital, Sichuan University (approval number: 2022‐1273). This study has been registered with the Chinese Clinical Trial Registry on May 11, 2023 (Clinical trial number: ChiCTR2300071330, https://trialsearch.who.int/Trial2.aspx?TrialID=ChiCTR2300071330)

## Conflicts of Interest

The authors declare no conflicts of interest.

## Supporting information




**Table S1**: Protocol of drug preparation in pH test and lamp inspection experiments.
**Table S2**: Protocol of drug preparation in HPLC for in vitro experiments.
**Table S3**: Grouping of animals in the preclinical study.
**Table S4**: Righting reflex evaluation scale for rats.
**Table S5**: The assessment indictors of sedation in animal study.
**Table S6**: The MOAA/S scale for sedation level assessment in patients.
**Table S7**: Changes in the pH value of mixed drugs within 14 days in different groups.
**Table S8**: Comparison of sedative and adverse reactions between two groups in clinical study.
**Table S9**: Comparison of satisfaction during induction period between two groups in clinical study.
**Figure S1**: Kaplan–Meier event‐free estimates of paresthesia. The time window for paresthesia occurrence in both groups was 40–60 s after drug administration.

## Data Availability

The data presented in the article may be requested by consulting the corresponding author with reasonable request.
