# Supplementary material for: Premixed Lidocaine With Fospropofol Disodium for Safety and Clinical Evaluation Regarding Paresthesia Upon Fospropofol Disodium Injection: A Preclinical Experimental Study and a Randomized Controlled Trial
Source: MedComm (2020). 2026 Mar 18;7(4):e70670. doi: 10.1002/mco2.70670 (PMC13042614; doi:10.1002/mco2.70670)
Supplement: Supplementary file 1 — Table S1: Protocol of drug preparation in pH test and lamp inspection experiments. Table S2: Protocol of drug preparation in HPLC for in vitro experiments. Table S3: Grouping of animals in the preclinical study. Table S4: Righting reflex evaluation scale for rats. Table S5: The assessment indictors of sedation in animal study. Table S6: The MOAA/S scale for sedation level assessment in patients. Table S7: Changes in the pH value of mixed drugs within 14 days in different groups. Table S8: Comparison of sedative and adverse reactions between two groups in clinical study. Table S9: Comparison of satisfaction during induction period between two groups in clinical study. Figure S1: Kaplan–Meier event‐free estimates of paresthesia. The time window for paresthesia occurrence in both groups was 40–60 s after drug administration. [file MCO2-7-e70670-s001.pdf]

Pre-mixed lidocaine with fospropofol disodium for safety and clinical evaluation regarding paresthesia upon fospropofol disodium injection: A preclinical experimental study and a randomized controlled trial

Bo Jiao[1,2,#], Xiaolin Xu[1,2,#], Ying Cui[1,2#], Caiyi Yan[1,2], Liyun Deng[1,2], Dequan Zhong[1,2], Qing Yang[3], Jiqian Xu[1,2], Yi Liu[1,2], Xiaohui Sun[1,2], Mengqian Xu[1,2], Tao Liu[1,2], Hui Xu[1,2], Xuejiao Tang[1,2], Xiaoqin Luo[4], Peng Liang[1,2], Jin Liu[1,2,*], Chan Chen[1,2,*]

[1] Department of Anesthesiology, West China Hospital, Sichuan University, Chengdu, Sichuan, China.

[2] Laboratory of Anesthesia and Critical Care Medicine, National-Local Joint Engineering Research Centre of Translational Medicine of Anesthesiology, West China Hospital, Sichuan University, Chengdu, Sichuan, China.

[3] Department of Anesthesiology, Huaihe Hospital of Henan University, No. 8, Baobei Road, Gulou District, Kaifeng 475000, China.

[4] Department of Anesthesiology, Ganzi Tibetan Autonomous Prefecture People's Hospital, Kangding 626000, Sichuan Province, China

*Corresponding author: Chan Chen, M.D., Ph.D.; Jin Liu, M.D., Ph.D.

Department of Anesthesiology and Laboratory of Anesthesia and Critical Care Medicine, National-Local Joint Engineering Research Centre of Translational Medicine of Anesthesiology, West China Hospital, Sichuan University, Chengdu, Sichuan, China, 610041. Email: chenchan@scu.edu.cn (Chan Chen); scujinliu@foxmail.com (Jin Liu)

Tel./fax: +862885423593.

Telephone: +86-18980606260.

[#] These authors contributed equally to this work.

Table of Contents

Supplementary Tables 3

Table S1. Protocol of drug preparation in pH test and lamp inspection experiments.................3

Table S2. Protocol of drug preparation in HPLC for in vitro experiments................................3

Table S3. Grouping of animals in the preclinical study...4

Table S4. Righting reflex evaluation scale for rats..4

Table S5. The assessment indictors of sedation in animal study..4

Table S6. The MOAA/S scale for sedation level assessment in patients 5

Table S7. Changes in the pH value of mixed drugs within 14 days in different groups............5

Table S8. Comparison of sedative and adverse reactions between two groups in clinical study 6

Table S9. Comparison of satisfaction during induction period between two groups in clinical study 7

Supplementary Figure 7

Figure S1. Kaplan–Meier event-free estimates of paresthesia 7

Table S1. Protocol of drug preparation in pH test and lamp inspection experiments.

Group name	Drug composition		
	fospropofol	2% lidocaine	0.9% normal saline
5 LD	/	0.5 ml	1.5 ml
7.5 LD	/	0.75 ml	1.25 ml
FP	100 mg	/	2 ml
5 LFP	100 mg	0.5 ml	1.5 ml
7.5 LFP	100 mg	0.75 ml	1.25 ml

Abbreviations: 5 LD group 5 mg/ml lidocaine, 7.5 LD group 7.5 mg/ml lidocaine, FP group 50 mg/ml fospropofol, 5 LFP group 5 mg/ml lidocaine plus 50 mg/ml fospropofol, 7.5 LFP group 7.5 mg/ml lidocaine plus 50 mg/ml fospropofol.

Table S2. Protocol of drug preparation in HPLC for in vitro experiments.

Group name	Drug composition		
	fospropofol	2% lidocaine	0.9% normal saline
0.5 LD	/	25 μl	975 μl
0.75 LD	/	37.5 μl	962.5 μl
0.1 FP	5 mg	/	1000 μl
0.5 LFP	5 mg	25 μl	975 μl
0.75 LFP	5 mg	37.5 μl	962.5 μl

Abbreviations: 0.5 LD group 0.5 mg/ml lidocaine, 0.75 LD group 0.75 mg/ml lidocaine, 0.1 FP group 5 mg/ml fospropofol, 0.5 LFP group 0.5 mg/ml lidocaine plus 5 mg/ml fospropofol, 0.75 LFP group 0.75 mg/ml lidocaine plus 5 mg/ml fospropofol. HPLC, High-performance liquid chromatography.

Table S3. Grouping of animals in the preclinical study.

Group name	Treatments†
FP	50 mg/ml fospropofol was administered
5 LFP	A drug combination containing 5 mg/ml lidocaine and 50 mg/ml fospropofol was administered
7.5 LFP	A drug combination containing 7.5 mg/ml lidocaine and 50 mg/ml fospropofol was administered

Abbreviations: FP group 50 mg/ml fospropofol, 5 LFP group 5 mg/ml lidocaine plus 50 mg/ml fospropofol, 7.5 LFP group 7.5 mg/ml lidocaine plus 50 mg/ml fospropofol.

† Dosage calculated by fospropofol: 82 mg/kg, iv.

Table S4. Righting reflex evaluation scale for rats.

Score	Righting reflex behavior
1	Complete disappearance
2	Uncoordinated activity, recovery not within 30 seconds
3	Uncoordinated activity, recovery within 30 seconds
4	Coordinated activity, recovery within 10 seconds
5	Complete wakefulness

Table S5. The assessment indictors of sedation in animal study.

Indictors	Behavior
Sedation onset time	The disappearance time of the righting reflex in rats (righting reflex score = 1)
Sedation duration	From the disappearance of the righting reflex to the recovery of the righting reflex (righting reflex score = 3)
Recovery time	from recovery of righting reflex to restoration of coordinated activity (righting reflex score = 4)

Table S6. The MOAA/S scale for sedation level assessment in patients.

Score	Behavior
0	No response to a loud auditory stimulus or mild prodding or shaking
1	Response only after a loud auditory stimulus or mild prodding or shaking
2	Response to a normal voice but appears drowsy
3	Response to a normal voice but alert
4	Lethargic response to a normal voice
5	Fully alert, no sedation

Abbreviations: MOAA/S, Modified Observer's Assessment of Alertness/Sedation

Table S7. Changes in the pH value of mixed drugs within 14 days in different groups.

Time	5 LD	7.5 LD	FP	5 LFP	7.5 LFP
0 h	5.90	5.83	8.93	7.75	7.63
0.5 h	5.96	5.86	8.77	7.74	7.64
1 h	6.02	5.89	8.70	7.74	7.64
2 h	6.02	5.83	8.79	7.70	7.59
4 h	5.94	5.81	8.68	7.70	7.59
8 h	6.05	5.89	8.58	7.70	7.59
12 h	6.07	5.87	8.57	7.70	7.58
24 h	6.10	5.91	8.45	7.69	7.58
48 h	6.16	5.93	8.39	7.69	7.59
72 h	6.15	5.95	8.31	7.69	7.58
4 d	6.14	5.96	8.30	7.69	7.58
5 d	6.20	6.14	8.28	7.68	7.58
6 d	6.17	6.18	8.24	7.67	7.58
7 d	6.19	6.18	8.23	7.69	7.58
14 d	6.31	6.25	8.13	7.65	7.55

Abbreviations: 5 LD group, 5 mg/ml lidocaine group; 7.5 LD group, 7.5 mg/ml lidocaine group; FP group, 50 mg/ml fospropofol group, 5 LFP group, 5 mg/ml lidocaine plus 50 mg/ml fospropofol group; 7.5 LFP group, 7.5 mg/ml lidocaine plus 50 mg/ml fospropofol group.

Table S8. Comparison of sedative and adverse reactions between two groups in clinical study. Data are given as n (%), and mean [standard deviation].

Outcomes	FP+LD group (n = 36)	FP+NS group (n = 34)	*P*-value
Time to achieve a MOAA/S ≤ 1 after administration, s	238.9 [105.9]	261.5 [111.5]	0.387
Time to loss of eyelash reflex after administration, s	227.6 [103.2]	247.2 [107.7]	0.440
Time to intubation, s	371.8 [116.0]	400.8 [135.2]	0.338
Time to start anesthesia maintenance, min	13.4 [5.1]	13.6 [4.3]	0.881
Injection pain	0 (0)	1 (2.9%)	0.486
Hypotension during anesthesia induction (n = 68)[†]	n = 35<br>13 (37.1%)	n = 33<br>12 (36.4%)	0.947
Bradycardia during anesthesia induction (n = 69)[‡]	n = 35<br>1 (2.9%)	0 (0)	> 0.990
Other complications (n = 72)	1 (2.8%)	n = 36<br>3 (8.3%)	0.614

Abbreviations: FP, fospropofol; LD, lidocaine; NS, normal saline; MOAA/S, Modified Observer's Assessment of Alertness/Sedation Scale.

[†] Due to equipment issues, partial blood pressure data were not collected during the observation period in two patients (one is in the lidocaine group, another in the control group).

[‡] one patient in the lidocaine group was excluded due to the heart rate data missing.

Table S9. Comparison of satisfaction during induction period between two groups in clinical study. Data are given as n (%).

Outcomes	FP+LD group (n = 36)	FP+NS group (n = 36)	*P*-value
Patient satisfactory assessment			0.598
Very satisfied	24 (66.7%)	22 (61.1%)	
Satisfied	12 (33.3%)	12 (33.3%)	
Neutral	0 (0)	2 (5.6%)	
Anesthesiologist satisfactory assessment			0.409
Very satisfied	5 (13.9%)	5 (13.9%)	
Satisfied	25 (69.4%)	24 (66.7%)	
Neutral	6 (16.7%)	4 (11.1%)	
Dissatisfied	0 (0)	3 (8.3%)	

Abbreviations: FP, fospropofol; LD, lidocaine; NS, normal saline.

Figure S1. Kaplan–Meier event-free estimates of paresthesia. The time window for paresthesia occurrence in both groups was 40-60 seconds after drug administration.

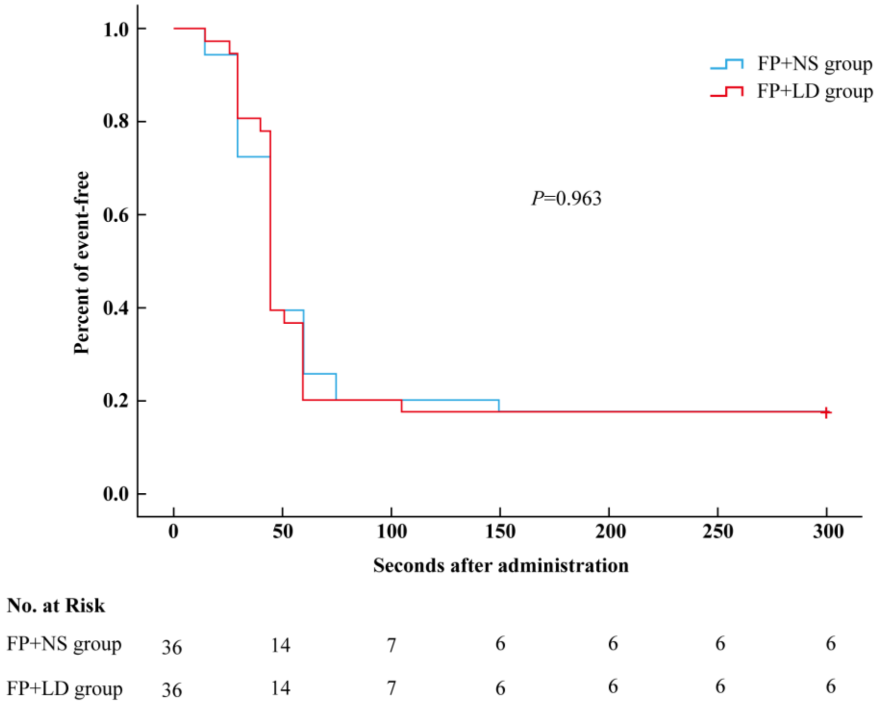

Abbreviations: FP, fospropofol; LD, lidocaine; NS, normal saline.
